# On Some Points Connected with Cholera

**Published:** 1889-08-31

**Authors:** 


### On Some Points Connected with Cholera.

*(Continued from page 322.)*

### A MODERN THEORY.

The theory of water being the vehicle by which cholera is conveyed has obtained the greatest number of supporters. In 1849 Dr. Snow first pointed out this probability; and in 1854 occurred the celebrated Broad-street poisoning case, which was investigated by a committee, whose report contains evidence convincing to many of cholera being disseminated by drinking water contaminated by cholera fœcal matter. An occurrence adding much strength to the water theory was as follows: A Mr. Eley, having business in Broad-street, returned every evening to his house at Hounslow, taking with him for the use of his mother and sister a bottle of Broad-street pump water. Both these ladies suffered from cholera, although there was no disease in the neighbourhood. In India, in the Hooghly, several P. and O. ships were moored close together. One vessel having something wrong with the water-tanks, was supplied with water by "bheestees" from the shore, whereupon twelve cases of cholera occurred, while the other ships remained free. There are numerous places where a supply of pure water has been followed by exemption from, or by diminution of, cholera. Bombay may be mentioned as a city in which cholera has been less constant since the wells were closed, and a water supply procured from the distant hills. Madras may also be mentioned, where a similar procedure has been followed by a similar result. But it is useless multiplying instances, for the evidence has become almost overwhelming of the communicability of cholera by water.

Our idea of cholera is as follows: There is an unknown atmospheric condition occurring more frequently in Eastern than in Western countries, which, meeting with certain but unknown conditions of matter, presenting most usually in unsanitary localities, generally an invisible, impalpable, imponderable, chemically and microscopically unrecognisable germ or poison, which may be conveyed in all directions by human beings, by clothing, by cholera excrete, by water, by food, by insects, especially flies, or through the atmosphere to an undiscovered extent; probably in a more certain manner, and in a more virulent form if favourable atmospheric conditions exist. These ideas do not credit Bengal with being the birthplace and home of cholera. They are opposed to the assumption of cholera being a malady which must spread in a definite direction from east to west, and arrive at a certain place at a certain date, and they do not limit the mode of dissemination to one particular channel. On the contrary, they assert the *de novo* origin of cholera under favouring circumstances in any country. They are based on the belief that the spread of cholera in a definite direction from east to west is a mistaken notion, arising from false reasoning on the fact of cholera epidemics being most frequent in the East. They are further based on the belief that Western cholera epidemics would occur almost the same if there were no Eastern epidemics. It is submitted that because cholera occurs in 1882 (or any other year) in Bengal, the occurrence of cholera in Europe in 1885 (or any other year) should not be regarded as the sequence of the Indian epidemic. Had similar researches been made into cholera as it affects countries east of India, there would be similar reasons for asserting an eastern progress of the malady. But countries east of India are less known, and attention has not been directed so much to the East as to the West. All this is more in accordance with the general and local history of cholera than the theories which are promulgated from the general while ignoring the local history of the disease. There are instances of the outbreak of cholera when no explanation is admissible, except that of *de novo* origin, while the communication of the malady by human intercourse, by clothing, by some varieties of merchandise, by water, by food, by insects, or through the atmosphere, explains the origin of attacks which are otherwise unexplainable; and such explanation appears as certain as the exact sciences.

Some of the mystery and obscurity which attaches to cholera results from it having been regarded since 1832, in Europe, and since 1817, in India, as a new disease. But Macpherson, in his "Annals of Cholera," has satisfactorily shown that cholera is one of the most ancient diseases of which distinct descriptions exist. In support of the view of the antiquity of the malady in the East, there is the fact that the ancient Sanskrit writers were apparently well acquainted with cholera. In the Midan of Sushruta there is a description of a disease (formerly mentioned) termed "Vishuchuka," meaning purging, vomiting, and fever; while in other old Hindoo medical works we have "jioat anteshan," meaning fever and incessant purging. There is also the Chinese name "Ho eonan," and the Arabic "duan." Then there is the significant fact that all the different languages of the peninsula of India have a time-honoured name for the disease. The Portuguese certainly found cholera in India soon after their arrival at Goa in 1500, and it was recognised by the Madras Medical Board reports long previous to 1817. In Europe, there is reason to believe that one or other of the different phases of cholera occurred, and was described under various names, long before the reputed first epidemic of 1832. Hippocrates and Galen even described a malady very similar to cholera. And anyone reading Van Heyden's description of a disease in 1643 cannot doubt that it was cholera. It is true that some of the writings on which reliance has been placed to prove the antiquity of the disease do not always describe the malady marked by suppression of urine, rice-water stools, and collapse, which in the present day is usually, and, as we think, erroneously held to constitute cholera. But this is only in accordance with the very numerous variations of the disease, as is sufficiently demonstrated by the numbers of names it has been called in modern days. Moreover, cholera is a term of recent origin, and a very bad term, too, for cholera really signifies the flow of bile, while the want of bile is usually one of the prominent characteristics!

Although we do not know the precise cause of cholera, ignorance of predisposing causes is much less. For example, we know that all places visited by epidemics of intensity have usually features in common—viz., overcrowding, low-lying situation on alluvial soil, and defective sanitation. In most countries it will be found there are localities more or less practically exempt from cholera, and such places partake but little of the objectionable features noticed. Geological conditions alone do not seem to exert much influence, for we have seen cholera on granite rocks, on black soil, and on sandy deserts. As regards elevation there is no doubt that mountainous districts are less conducive to cholera. Still, however, the disease has occurred on Indian hill stations up to an elevation of 7,330ft.

Season, again, is a predisposing cause, and throughout India, as a general rule, cholera reaches its maximum in July, or at the most hot and damp period of the year. Want is a strong predisposing cause, as sufficiently evidenced by the number of cholera cases during seasons of scarcity in India. Fatigue must also be regarded as a predisposing cause. Persons in India coming off a long journey are especially liable to cholera, particularly pilgrims, who, on their journeys to and from holy shrine, also often suffer from want. Fear has already been mentioned as predisposing. Worry and anxiety may also be added. It is well known that anxiety of mind conduces to the invasion of most maladies, from a common cold upwards, and it especially predisposes to cholera. It is well known that any sudden shock often causes an action of the bowels, and that anxiety may produce a chronic looseness even when there is no cholera about. Of this we are quite certain, that persons who are depressed and alarmed are most likely to become the victims of cholera, that in the presence of a cholera epidemic panic intensifies its force, and in many instances developes apparently simple diarrhœa into cholera. Yet, as mentioned before, great fear is unreasonable, for in the worst epidemics immunity is the rule. Chill, again, is a fertile predisposing cause. It has already been observed that cholera most frequently occurs in the early morning hours, when the temperature of the air is lowest. Hence the necessity of caution against chill during epidemics. Another predisposing cause is intemperance. Instances of the disease developing itself during the depression following a debauch are numerous, and this should be guarded against, especially in military life. But during an epidemic it is not safe entirely to cease the use of spirits when indulgence in them has been habitual. Soldiers in India are aware of this, and not unfrequently say they cannot leave off drinking till the epidemic has passed away, for if they do they are sure to get the disease. Brandy-drinkers are said to be most liable. But any kind of alcohol has a special affinity for the nervous centres, and alcohol can itself excite vomiting and purging. It therefore appears particularly calculated to induce an especial predisposition to a malady of which nervous prostration, purging, and vomiting are the essential characteristics.

*(To be continued.)*

